# Miscellaneous

**Published:** 1842-07-01

**Authors:** 


### Miscellanies.

### Sympathetic Paralysis.

Dr. Zabriskie, an American physician, has related cases of this kind in a late Number of our esteemed contemporary, the American Journal of Medical Sciences, some of which we shall greatly condense, being rather more minutely detailed than appears to be necessary on this side of the Atlantic.

*Case* 1.—This was communicated to the author by Dr. Kissam of Jamaica. Mr. Williamson, of spare habit, had laboured under kidney disease for more than a year. He was unable to walk erect—pain in the region of the kidneys and ureters—perspiration of urinous odour—sallow complexion—tongue furred—breadth fetid—bowels costive—frequent micturition—red sediment in the urine. He was treated with mild medicines, and became much improved, and was able to walk about, and even attend a little to his farm. Six months afterwards (June 8, 1840) the doctor was summoned, and found the patient labouring under paraplegia—at least of motion, sensation remaining. Any attempt to move the head induced convulsions and startings in the paralyzed limbs. Excessive pain existed in the lower extremities. Through the catheter, pus, gravel, and mucus were discharged with the urine. The region of the right kidney was enlarged and tense. Appetite was nul—tongue foul and brown—perspiration excessive—stools not involuntary. He lingered out till the 10th of July, when he died.

As no dissection took place, the case, though interesting in itself, has no claim to be placed in the category of "sympathetic" paralysis. There can scarcely be a doubt that, in this instance, there was caries or other affection of the spine producing paraplegia.

Mr. Abernethy has related cases of hemiplegia supervening on hepatitis, and removed when the liver affection gave way. In some cases, where death took place, no disease of the spinal marrow or brain could be detected. But because the structure of these parts does not present visible or tangible alterations from the normal condition, are we to argue from this that no change occurred? *Functional disorders* may induce death, and leave no cognizable trace of the nature or cause of the fatal event.

*Case* 2.—Miss L. aged 16 years, was seized with acute hepatitis in the month of April, 1831. The symptoms were characteristic, but did not give way, and the disease became chronic, accompanied by amenorrhœa, and severe pain in the loins—numbness of the right leg—and ultimately paralysis of that extremity *every afternoon*. In the morning apyrexia, the power of motion, and also sensation returned. Cupping, blistering, and even issues were used, but without any benefit. Twenty leeches were applied to the region of the liver, which relieved the pain there, and removed the diurnal fever, together with the paralysis. This was a fair case of paralysis sympathetic of liver disease.

*Case* 3.—*Enteritic Paralysis.*—Thomas Lynch, ætat. 28, sanguine temperament and sober habits, entered the Alms-house in June, 1835, with complete paralysis of the lower extremities, as well as of the sphincters of the bladder and rectum. There was weakness with tenderness in the loins, and constant discharge of bloody mucus from the bowels, with tenderness and pain in the course of the colon. The colitis was treated by calomel, opium, and Dover's powder, while the warm bath, frictions, liniments, leeches, blisters, &c. &c. were prescribed for the paralysis, without any benefit. As the dysentery and abdominal pain continued leeches were applied to the epigastrium. The next day the patient could move his legs. "From this time the dysentery ceased, and the sphincters regained their power. The leechings were occasionally repeated, and, in a few weeks the patient was able to walk about the wards. This was an instructive case.

*Case* 4.—An old lady, aged 78 years, delicate and dyspeptic, awoke on the

20th August, with much stupor, and sense of great weakness. These increased for two days, when she appeared heavy and dull, with red eyes—apparent inability to raise the eye-lids—flow of saliva from the mouth—dysphagia—quickened pulse—white tongue. On the 24th there was complete paralysis of the left side. The fever appeared paroxysmal, and therefore quinine was prescribed. There were occasional improvements and exacerbations for several days, when recovery began, and progressively increased. "This, says our author, was evidently a case of intermittent paralysis, having an evening exacerbation, and being more severe every second day, the paralysis depending on the same cause as the fever."

*Case* 5.—Ann Masters, aged 14, was admitted into the Alms-house in 1837, with chronic diarrhœa, for which many remedies had been tried in vain. The lower extremities became gradually weak, and at length paralyzed. The sphincters participated. She died, and, on dissection the colon was found much inflamed, but the chief seat of the disease was in the jejunum, which was in a state of high inflammation, the mucous membrane being pulpy and disorganized. No disease could be detected in the spine or spinal marrow; but the nerves of the lower extremities were greatly wasted in size. The other viscera were sound.

*Case* 6.—Mrs. V. S. was seized with a bilious fever, after accouchment, and this was succeeded by dysentery. This last was attended with a morning chill and an evening re-action. During the pyrexia there were tonic spasms of various muscles, both in the extremities and in other parts. Twenty grains of calomel were given internally, and sinapisms were applied to the feet and spine. Next day it was found that the calomel had changed the appearance of the stools. A blister to the epigastrium, and two grains of calomel with five of Dover's powder, were given every three hours. In three days, the mouth became affected and the spasms and dysenteric symptoms subsided. Some months afterwards this woman was again seized with dysentery: but, instead of the spasms, she had now numbness of the lower limbs with nearly total loss of power in them. She was entirely helpless in the arms; but could swallow and speak without difficulty. There were no cerebral symptoms, and the sphincters retained their power. The lower dorsal vertebræ were tender. Twenty-five grains of calomel were given to her, with one of opium. A sinapism to the epigastrium. This was followed by dark-coloured evacuations. An ounce of castor-oil, which acted freely. A grain of calomel with ten of Dover's powder was given every four hours. Next day the dysentery was relieved, and the paralytic symptoms diminished. The same powders continued. She quickly recovered.

"The reasons for believing this species of paralysis as sympathetic of enteritis may be briefly summed up as follows:—

1. The inflammation always precedes the paralysis, and often for some time. This took place in Dr. Waddel's case, in all the cases of Mr. Abernethy, and in all the cases observed by myself, the diarrhœa preceded the paraplegia.

2. From the absence of all morbid appearances upon dissection, the nervous apparatus appearing sound.

3. From the inutility of all remedies applied to the spine, to the brain, or to the general nervous system.

4. The remedies which gave most relief were those which relieved the inflammatory symptoms.

The diagnosis of this form of paraplegia may be attended with difficulty. But it may be distinguished from cerebral paralysis by the absence of all symptoms of diseased brain. From the paralysis arising from a disease of the spine by the absence of the severe pain, and tenderness upon pressure in a diseased state of the vertebræ, by the existence of visceral inflammation, by the effect of remedies used for enteritis, and by the gradual manner of its approach.

The practice which I have found most successful is that calculated to subdue the inflammatory symptoms.

Bleeding general and local to subdue the phlogosis, accompanied by evacuants, followed by mercurials when these symptoms abate, to change the morbid secretions, and, by counter-irritation, to divert the inflammation. When the inflammatory symptoms have somewhat subsided, strychnine forms an excellent and valuable remedy in paraplegia."—*American Journ. Med. Sciences, Oct.* 1841.

---

### Twelfth Annual Report of the Belfast District Lunatic Asylum. March 1842.

The following sensible passage on this well-conducted Asylum, bears on a "*vexata quæstio*" of the present day.

### RESTRAINT OF PATIENTS.

"The cases in which instrumental restraint,—by the imposition of a strait-waistcoat on the person, or muffs on the hands,—was obliged to be had recourse to, from time to time, during the past year, were confined to about four, out of the entire number of inmates; and these almost exclusively amongst the females, some of whom were so uncontrollably violent in their general conduct, as well as destructively inclined, that all other methods were found totally inefficient, as moral agents, in repressing their disposition to commit acts of outrage, personal and otherwise. One of the females, in particular, for several months past, has been most unconquerable in destructive and turbulent propensities, by breaking windows, doors, and locks; stripping off and tearing her clothes, striking the attendants, &c., &c.; neither persuasions, or threats, or even the offering of a reward, for ordinary good conduct, when tried at large, having the least effect in deterring her from the commission of such acts as the above; and not only does she behave in this insubordinate manner herself, but, in her morbid proneness to mischief, endeavours to make other patients equally unruly, and to a certain extent succeeds in doing so; thus keeping up, day and night, at intervals, a harassing state of excitement and riot in the division to which she belongs; but these interruptions, in the entire personal freedom, general order and comparative quietude which ordinarily prevail in the establishment, are, after all, but of small moment, and wonderfully few, when we consider the deplorable nature of the mysterious malady its unhappy inmates are the subjects of.

---

### Hanwell Lunatic Asylum.

We have much pleasure in recording the recent delivery of a series of clinical lectures in this Institution, with admission to what may be termed the practice of the Asylum. Great credit is due both to Dr. Conolly for his exertions in bringing it about, and to the Visiting Justices for their boldness and liberality in consenting to the experiment, notwithstanding the numerous objections which were urged against it, and the prejudices which it had to encounter. It must be a source of great satisfaction to all concerned, to find that the experiment has succeeded so well, that no excitement appears to have been produced amongst the patients by the visits of the students, whilst the students have had an opportunity of acquiring, for the first time, much valuable information upon a subject, the study of which has hitherto been attended with so many difficulties.

The plan adopted with regard to the admission was, to give to each of the principal Metropolitan hospitals, the privilege of sending one of their more advanced pupils; thus at once reducing the number within the necessary limits, and by having only senior pupils present, rendering it unnecessary to occupy any valuable time with the more elementary parts of the subject.

We shall refrain from noticing more particularly the lectures themselves at present, as we are not without hopes that Dr. Conolly may be induced soon to publish them in some form or other, an address having been presented to him to that effect at the last lecture.

